# Profiles of patient and tumour characteristics in relation to health-related quality of life after oesophageal cancer surgery

**DOI:** 10.1371/journal.pone.0196187

**Published:** 2018-04-30

**Authors:** Poorna Anandavadivelan, Anna Wikman, Asif Johar, Pernilla Lagergren

**Affiliations:** 1 Surgical Care Science, Department of Molecular medicine and Surgery, Karolinska Institutet, Karolinska University Hospital, Stockholm, Sweden; 2 Reproductive Health, Department of Women’s and Children’s Health, Uppsala University, Uppsala, Sweden; CANADA

## Abstract

Strong deterioration in health-related quality of life (HRQOL) is a major concern in a sub-group of long-term oesophageal cancer survivors. This study aimed to identify potential clustering of patients and tumour variables that predicts such deterioration. Patient and tumour variables were collected in a prospective cohort of patients who underwent surgery for oesophageal cancer in Sweden 2001–2005. Latent cluster analysis identified statistically significant clustering of these variables. Multivariable logistic regression adjusted for age, BMI, tumour stage and marital status was used to determine odds ratios (ORs) with 95% confidence intervals (CIs) between patient profiles and HRQOL at 3 and 5 years from surgery. Among 155 included patients at 3 years, three patient profiles were identified: 1) ‘reference profile’ (males, younger age, employed, upper secondary education, co-habitating, urban dwellers, adenocarcinoma and advanced tumour stage) (n = 47;30%), 2) ‘adenocarcinoma profile’ (middle age, unemployed/retired, males, low education, co-habitating, adenocarcinoma, advanced tumour stage, tumour in lower oesophagus/cardia, and co-morbidities (n = 79;51%), and 3) ‘squamous-cell carcinoma profile’ (unemployed/retired, middle-age, males, low BMI, urban dwellers, squamous-cell carcinoma, tumour in upper/middle oesophagus (n = 29;19%). These profiles did not differ regarding most HRQOL measures. Exceptions were the squamous-cell carcinoma profile, reporting more constipation (OR = 5.69; 95%CI: 1.34–24.28) and trouble swallowing saliva (OR = 4.87; 95%CI: 1.04–22.78) and the adenocarcinoma profile reporting more dyspnoea (OR = 2.60; 95%CI: 1.00–6.77) and constipation (OR = 3.31; 95%CI: 1.00–10.97) compared to the reference profile. Three distinct patient profiles were identified but these could not explain the substantial deterioration in HRQOL observed in the sub-sample of survivors.

## Introduction

Oesophageal cancer has an increasing incidence [1, 2] and poor prognosis (5 year survival <20%) [1]. The curative treatment typically includes neo-adjuvant chemotherapy or chemoradiotherapy followed by surgery (oesophagectomy) [3]. Oesophagectomy is more extensive than most other surgical procedues and is often (30–60%) associated with severe post-operative complications [4]. The survivorship in patients having undergone curative treatment is characterised by long-lasting deterioration in health-related quality of life (HRQOL) and weight loss corresponding to malnutrition [5, 6]. We have previously found that, an important sub-group of patients deteriorate dramatically in most HRQOL aspects between 6 months and 5 years from surgery despite absence of tumour recurrence [7]. A potential strategy to improved recovery would be to early on identify patients who are at an increased risk for such strong deterioration in HRQOL. Further, if HRQOL measures can detect such fluctuations in HRQOL, these can also reasonably be used to monitor the effect of potential interventions to improve HRQOL [8]. Thus, it is imperative to identify a risk profile of patient characteristics that can predict poor recovery in HRQOL. Such identification could ideally be used in clinical practice to prompt early postoperative interventions and heightened attention during the follow-up of these patients. Therefore, we conducted a population-based cohort study with the aim to identify risk factor profiles that predict severe deterioration in HRQOL following surgery for oesophageal cancer.

## Materials and methods

### Design

This was a nationwide prospective cohort study comprising almost all patients (90%) operated on for cancer of the oesophagus or gastro-oesophageal junction in Sweden between April 2, 2001 and December 31, 2005, with at least 5-year follow-up of all participants in December 31, 2010. The study exposures were age, gender, body mass index (BMI), education, employment status, marital status, geographical location, histological type, tumour location, tumour stage, co-morbidities and the study outcome was HRQOL following treatment. Written informed consent was obtained from all participants and the study was approved by the Regional Ethical Review Board in Stockholm, Sweden.

### Study exposures

The clinical data collection has been described in detail elsewhere [9–11]. Briefly, clinical data at diagnosis were prospectively obtained from medical records using a pre-defined study form ensuring uniformity in data collection. All patients in the source cohort were linked to the Longitudinal Integration Database for Health Insurance and Labour Market [12] in September 2012 for information on marital status, education level, employment status and geographical location [12]. The registers were linked by means of the personal identity number, assigned uniquely to all Swedish residents. The selection of variables as exposures was based on previous research [11, 13–19] as listed below:

1. Age at time of operation—categorised as <60 years/60-74 years />74 years.
2. Gender—female/male.
3. BMI—Patients were asked to report their height and weight at operation. BMI was calculated as weight in kg / height x height in m^2^) and categorised as low (≤24.9)/high (≥25).
4. Education was categorised as nine year compulsory/upper secondary/higher education degree.
5. Employment—unemployed/employed.
6. Marital status—single/cohabitating.
7. Geographical location—rural/urban.
8. Histological type—squamous cell carcinoma/adenocarcinoma and dysplasia.
9. Tumour location—was categorised based on the Siewert classification [20] as lower oesophagus and cardia/upper and middle oesophagus.
10. Tumour stage—was classified according to the International Union Against Cancer as TNM staging systems, 6^th^ edition grouped into tumour stage I to IV [21]. The pathological TNM data (at surgery) were used to define tumour stage.
11. Co-morbidities including hypertension, angina, heart failure, chronic obstructive pulmonary disease, diabetes and kidney disease categorised as yes/no.

### Study outcomes

HRQOL was measured at 3 and 5 years after surgery using well-established questionnaires, developed and validated by the European Organisation for Research and Treatment of Cancer (EORTC). The QLQ-C30 (version 3.0) is a cancer-specific questionnaire consisting of 30 items examining five functional scales, three symptom scales, and one global quality of life scale [22]. The QLQ-OES18 is a disease-specific module including four symptom scales and six single items [23, 24]. Responses obtained from the HRQOL questionnaires were linearly transformed into scores between 0 and 100, according to the EORTC scoring manual [25]. For the global quality of life and functional scales, a higher score indicates better HRQOL whereas for symptom scales a higher value signifies more symptoms. A mean score difference (MSD) of HRQOL scores ≥10 between 6 months-3 years [26] and 6 months-5 years [7] was considered clinically moderately relevant, and an MSD of ≥20 was considered clinially strongly relevant based on pre-established cut-offs [27]. Patients’ responses to each HRQOL scale or single item were categorised as improved or stable (i.e. an improvement in MSD of ≥10 on a scale of 0 to 100, or <10 MSD when the score reaches the maximum score e.g., improvement from 95 to 100 mean scores) or deteriorated (i.e. a decrease in MSD of ≥10 on the 0 to 100 scale, or <10 MSD when the score reaches the minimum score e.g., deteriorating from 5 to 0 mean scores) based on the calculated MSD [7, 26].

### Statistical analysis

The statistical analyses were carried out separately for HRQOL at 3 years and 5 year after surgery. Latent class cluster analysis was used to identify underlying profiles of patients based on the study exposures presented above. This cluster analysis is a statistical model used to identify homogeneous and mutually exclusive profiles of patients within a patient population. The model with 3 profiles were selected on the basis of the minimum Bayesian Information Criterion[28]. Within each profile the predominant characteristics of the profile was identified using combination of Chi-square test and the most prevalent category. The most prevalent category within each characteristics was selected if the ratio of posterior-proportions for the most prevalent category was >1.5. Because missing data were rare (<2%), a complete case analysis was used.

A multivariable logistic regression analysis adjusted for sex, BMI, tumour stage and marital status was performed to assess the association of patient profiles, obtained from the Latent class cluster analysis, with the deteriorated or improved/stabilised group for each HRQOL scale, estimated as odds ratio with 95% confidence intervals. To address the issue with using some covariates in both models, we have assumed conditional independence between covariates (X) and quality of life scores (Q) given the latent class information (C) i.e., we assume that f(X, Q|C) = f(X|C)f(Q|C) [29]. SAS 9.4 (SAS Institute Inc) and PROC Latent class cluster analysis [30] procedure were used in the analysis.

## Results

### Patients

A total of 616 patients were recruited in the source cohort. Of these, 506 (82%), 211 (34%) and 153 (25%) patients were alive at 6 months, 3 years and 5 years, respectively. Among these survivors, 402 (79%), 178 (84%), and 141 (92%) answered the HRQOL questionnaires at 6 months, 3 years and 5 years. Thus, 155 (74%) patients who responded to both the HRQOL questionnaires at 6 months and 3 years were eligible for the 6 months to 3 years analysis and 117 (78%) were eligible for the 6 months to 5 years analysis and included in the study. Characteristics of the patients who were eligible for inclusion at 3 years and 5 years in this study are presented seperately in Table 1. Majority of patients at both follow ups were men, middle aged (60–74 years) and unemployed/retired. Most were co-habitating and lived in an urban location. The dominating tumour type was adenocarcinoma and dysplasia in the lower oesophagus and cardia. More than half of the patients had one or more co-morbidities. Characteristics of responders and non-responders were assessed and did not differ with statistical significance (data not shown).

**Table 1:** Characteristics at diagnosis of patients who responded to HRQOL questionnaires at 3 years and 5 years following surgery for oesophageal cancer.

Characteristics at diagnosis	Patients followed up for HRQOL			
	3 years (n = 155)		5 years (n = 117)	
	n	%	n	%
**Age**				
< 60 years (younger)	39	25	32	27
60–74 years (middle aged)	99	64	75	64
> 74 years (older)	17	11	10	9
**Gender**				
**Female**	33	21	24	20
**Male**	122	79	93	80
**BMI**				
≤ 25	77	50	52	44
> 25	78	50	65	56
**Education**				
Nine year compulsary	69	45	53	45
Upper secondary	62	40	44	38
Higher education degree	22	14	19	16
**Employment status**				
Employed	47	30	42	36
Unemployed/Retired	108	70	75	64
**Marital status**				
Single	50	32	31	26
Cohabitating	104	67	85	73
**Geographical location**				
Rural	60	39	45	38
Urban	95	61	72	62
**Histological type**				
Squamous cell carcinoma	38	25	29	25
Adenocarcinoma and dysplasia	117	75	88	75
**Tumour location**				
Lower oesophagus and cardia	132	85	100	85
Upper and middle oesophagus	23	15	17	15
**Tumour stage**				
0—I	61	39	53	45
II	51	33	36	31
III-IV	42	27	27	23
Co-morbidities*				
No	61	39	52	44
Yes	94	61	65	66

* Comorbidities include hypertension, angina, heart failure, chronic obstructive pulmonary disease, diabetes and kidney disease.

### Exposure profiles

**Fig 1:**
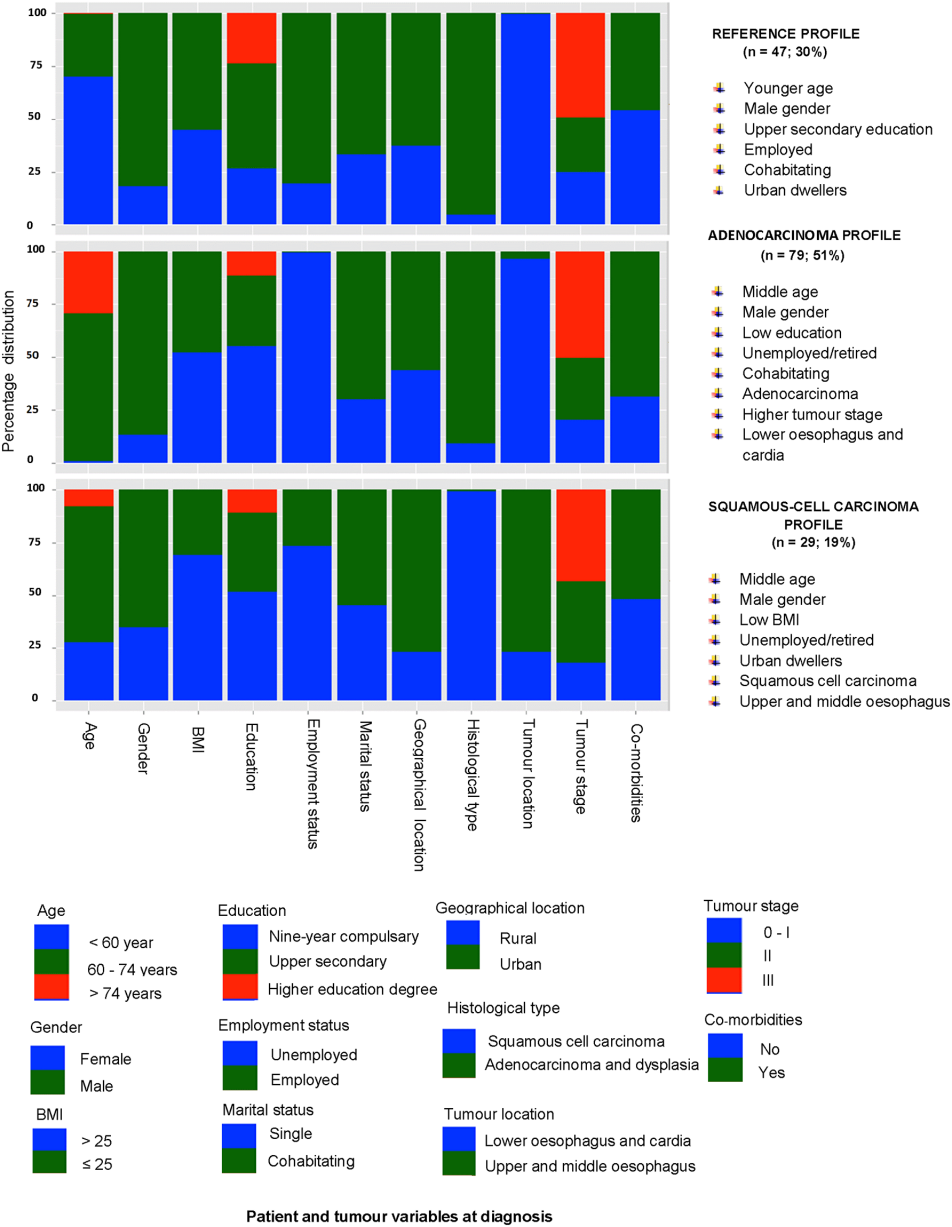
Proportion of socio-demographic, anthropometric and clinical characteristics at diagnosis among the reference, adenocarcinoma and squamous-cell carcinoma profiles created from latent class cluster analysis of 3-year survivors of oesophageal cancer following surgery.

The Latent class cluster analysis for the 3-year survivor group of 155 patients yielded three patient profiles presented in Fig 1.

1. The first profile included 47 patients (30%) and was characterised by males (82%), younger age (70%), employed (81%), upper secondary education (50%), co-habitating (67%) in urban areas (63%) with adenocarcinoma (95%)and tumour stage III-IV (49%). This was chosen as the ‘reference profile’ because the characteristics resembled a healthier population. This group was the reference group in the logistic regression analyses.
2. The second profile included 79 patients (51%) and comprised of middle age (70%), unemployed/retired, males (100%), low education (55%), co-habitating (70%), with adenocarcinoma (91%) of tumour stage III-IV (50%) located in the lower oesophagus /cardia (96%), and presence of co-morbidities (69%) at diagnosis as the predominant characteristics. It was labelled as the ‘adenocarcinoma profile’ to represent the most common histogical sub-type in this profile.
3. The third profile included 29 patients (19%) and was characterised by unemployed/retired (73%), middle-aged (64%), males (65%), low BMI (69%), urban dwellers (72%), with squamous cell carcinoma (99%) in the upper or middle oesophagus (77%). It was labelled ‘squamous-cell carcinoma profile’ to highlight the more common histological sub-type of this profile.

A similar profile distribution was observed for the 5-year survivor group (data not presented).

### Adenocarcinoma profile and risk of deterioration in HRQOL

**Fig 2:**
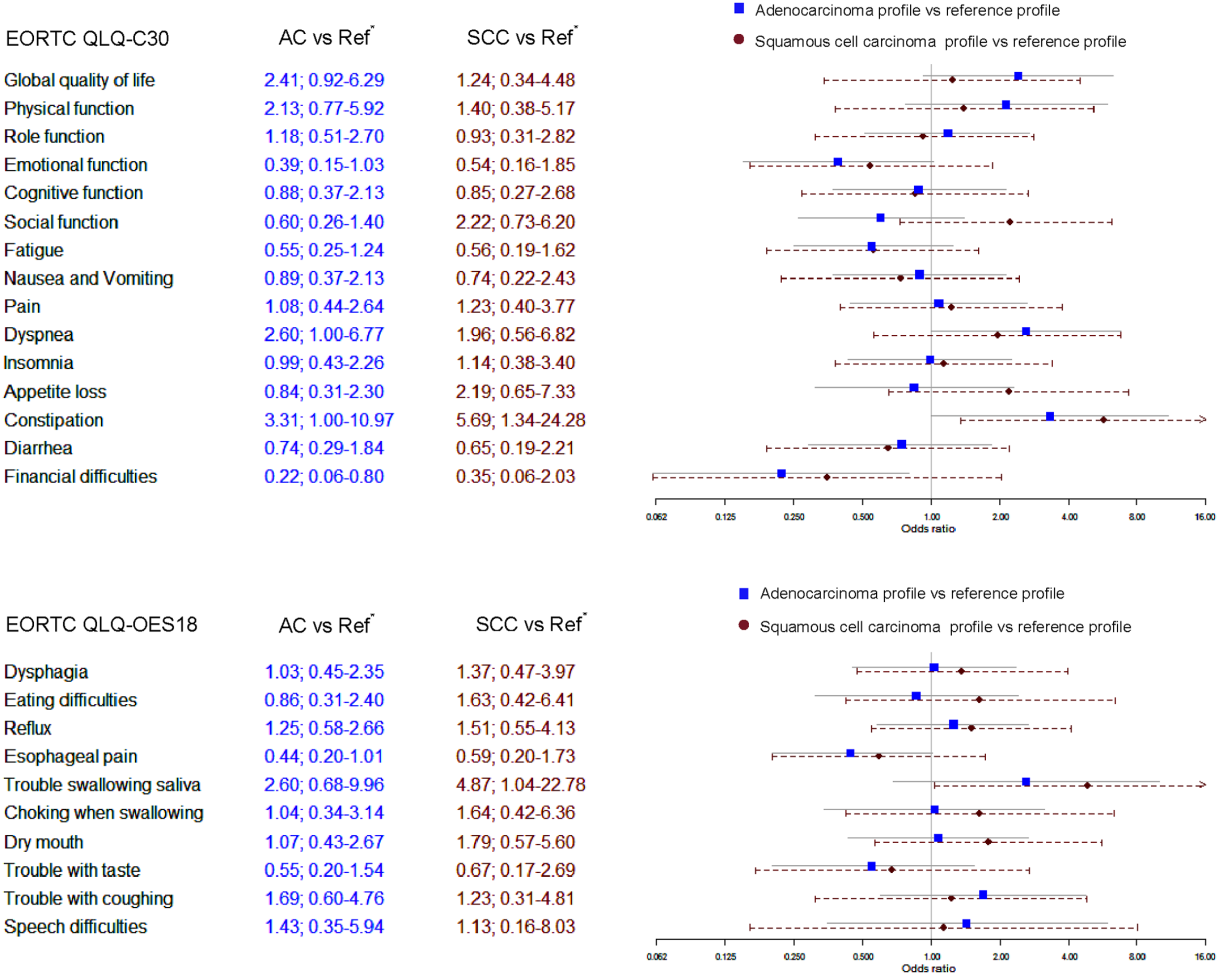
Graph illustrating association between the three patient profiles created from latent class cluster analysis and risk of deterioration in healthelated quality of life (HRQOL) aspects assessed by multivariable logistic regression analysis adjusted for age, BMI, tumour stage and marital status in patients who survived at least 3 years after surgery for oesophageal cancer. The blue squares represents odds ratio obtained from adenocarcinoma profile versus the reference profile and grey lines represent the confidence intervals. Red dots represent odds ratio obtained from the squamous-cell carcinoma profile versus the reference profile and dotted red lines represent confidence intervals. HRQOL was assessed at 6 months and 3 years from surgery using the European Organisation for Research and Treatment of Cancer questionnaires QLQ-C30 and QLQ-OES18. Scores obtained were converted into linear scale scores of 0 to 100 and mean score differences of ≥10 between scores at 6 months and 3 years were considered clinically moderately relevant and ≥20 as clinically strongly relevant. HRQOL of patients were also categorised as improved and stable/deteriorated for each aspect of HRQOL. Latent class cluster analysis of patient’s socio-demographic, anthropometric and clinical characteristics at diagnosis yielded three patient profiles: Reference profile: Younger age, male gender, upper secondary education, employed, cohabitating, urban dwellers. Adenocarcinoma profile: Unemployed/retired, male gender, low education, cohabitating, adenocarcinoma, higher tumour stage, lower oesophagus and cardia, co-morbidities present. Squamous-cell carcinoma profile: Unemployed/retired, male gender, low BMI, unemployed, urban dwellers, squamous cell carcinoma, upper and middle oesophagus. AC—Adenocarcinoma; SCC—Squamous–cell carcinoma; EORTC—European Organisation for Research and Treatment of Cancer; QLQ—Quality of life questionnaire; HRQOL—Health Related Quality of Life; BMI—Body mass index.

Patients in the adenocarcinoma profile had no statistically significant differences in global quality of life (OR 2.41; 95% CI 0.92–6.29) compared to the reference profile (Fig 2). Among the functional scales, physical function (OR 2.13; 95% CI 0.77–5.92) and role function (OR 1.18; 95% CI 0.51–2.70) did not differ statistically compared to the reference profile. Pain, dyspnoea and constipation from among the general symptom scales and items were associated with a possible worsening over time for the adenocarcinoma profile in relation to the reference profile, with statistical significance observed for dyspnoea (OR 2.60; 95% CI 1.00–6.77) and constipation (OR 3.31; 95% CI 1.00–10.97). Concerning the oesophageal specific symptoms and items (QLQ-OES18) 7 out of the 10 scales indicated no differences statistically among the adenocarcinoma profile (Fig 2).

### Squamous-cell carcinoma profile and risk of deterioration in HRQOL

Compared with the reference profile, patients in the squamous-cell carcinoma profile had statistically no differences in global quality of life (OR 1.24; 95% CI 0.34–4.48) (Fig 2). Similarly, physical function (OR 1.40; 95% CI 0.38–5.17) and social function (OR 2.22; 95% CI 0.73–6.20) were statistically not different than the reference group among the functional scales. Of the general symptom scales and items, for pain, dyspnoea, insomnia, appetite loss and constipation no differences were reported than the reference profile however constipation alone was statistically significant (OR 5.69; 95% CI 1.34–24.28). Among the oesophageal specific symptom scales and items, 8 out of 10 were non-different for the squamous-cell carcinoma profile than the reference profile but trouble swallowing saliva alone was worse with statistical significance (OR 4.87; 95% CI 1.04–22.78).

A separate analysis for the 5 year survivors was also performed and similar results were observed between the three profiles and HRQOL (data not shown).

## Discussion

This study identified three distinct patient profiles, but few statistically significant associations were observed between these profiles and postoperative changes in HRQOL measures. Patients belonging to the adenocarcinoma profile were seemingly more likely to worsen in constipation and dyspnoea after surgery for oesophageal cancer, while patients in the squamous cell carcinoma profile were more likely to deteriorate in constipation and trouble swallowing saliva.

The nationwide population-based prospective cohort design and high participation and response rate counteracted selection bias, and the use of well-validated questionnaires secured the quality of the HRQOL outcomes. Well-structured and scrutinous data collection from medical records and precise linkage to the Longitudinal Integration Database for Health Insurance and Labour Market register through the Swedish personal identity number ensured high accuracy of clinical and socio-demographic exposure data, respectively. A weakness was the self-reported data for BMI. However, self-reported pre-surgical body weight measures correlate well with objectively measured weight in patients with oesophageal cancer because weight loss is a central issue for these patients [31]. Co-morbidities were categorised only as present or absent which may be a limitation, however a previous study from the same data source found that specific pre-surgical co-morbidities did not worsen HRQOL at 6 months after surgery with the exception of those with diabetes who reported more fatigue that was clinically relevant however not statistically significant [13]. Confounding in observational studies cannot be entirely accounted for, yet by using latent class cluster analysis, its effect may be considerably reduced, since the model is well-equipped for accommodating similarities and dissimilarities between the profiles. As our aim was to identify a risk profile/profiles, that may exist due to multidimensional interactions of these risk factors (which is difficult to model using conventional logistic regression analysis) associated with deterioration in HRQOL, we applied a two-step approach (latent class cluster analysis followed by logistic regression analysis). This circular approach may have led to variance inflation and reduced the likelihood of finding significant odds ratios for the individual predictors in the models. Another weakness is the limited statistical power.

In addition, although tumour recurrence is associated with poor survival in patients operated for oesophageal cancer [32], the vast majority of patients have recurrence within the first 1–2 years of surgery [33–35]. It is likely that in patients who still develop later recurrence, such recurrence would result in deterioration in HRQOL. In this cohort, however, only 14 (9%) patients died from oesophageal/gastric cancer within 6 months of the 3-year assessment, and these deaths were equally distributed between the profiles (9%, 9%, and 10% in profiles 1, 2, and 3 respectively). Within 6 months after the 5 year assessment, 4 (3%) died from oesophageal/gastric cancer, again without major differences in distribution between the profiles (5%, 2%, and 5% in profiles 1, 2, and 3 respectively). Since deaths after the follow-up time-point were few and similarly distributed among the three profile groups, tumour recurrence should not influence the results in the present study. Further, one might also argue that post-operative complications are known to be the strongest risk factor for poor HRQOL and incomplete recovery [4]. However, the aim of the current study being identification of risk patients at the time of diagnosis/before treatment, we have not included post-operative complications in the model. If a certain cluster would have predicted poor HRQOL recovery, such information may have facilitated identification of risk patients at time of diagnosis/before treatment which in turn could have guided a tailored follow for such risk patients. Furthermore we see complication as a mechanism that influences the HRQOL and can be part of the causal pathway. Of course, changes in HRQOL could have also happened earlier than the 3-year assessment, with any potential effect having diminished by this time.

HRQOL measurement has emerged as a resourceful tool in assessing the disease and oncological treatment impact on patients’ lives [36]. However, the reasons still remain unknown for the earlier elucidated deterioration in post-surgical HRQOL among the same cohort of patients as the present study[7]. None of the three patient profiles in this study could explain the reason behind a notable deterioration in HRQOL at 3 years or 5 years of surgery despite cure. Other factors should explain the decline in HRQOL, and these need to be identified in future research. For example personality traits such as dispositional optimism have been strongly associated with better HRQOL in head and cancer survivors compared to pessimism [37].

Earlier literature shows evidence for associations of individual risk factors to deterioration in post-surgical HRQOL in oesophageal cancer patients [38–40]. Co-morbidities, advanced tumour stage (III—IV), squamous cell carcinoma histology, and proximal tumour location can increase the risk of poor HRQOL at 6 months following surgery [11]. Tumour stage [18] and type have been indicated as independent determinants of HRQOL [11, 19]. However, the studies discussed above have focused mainly on individual risk factors. Our study is, to the best of our knowledge, the first to investigate if a profile of patient characteristics is potentially associated with a decline in HRQOL in oesophageal cancer survivors.

The adenocarcinoma profile had statistically significant associations with worsenening symptoms of dyspnoea and constipation and the squamous-cell carcinoma profile with worsening symptoms of constipation and trouble swallowing saliva in this study. Both these profiles had statistically no differences compared to the reference group with several HRQOL aspects. A possible explanation for the statistically significant associations may be multiple testing which may have led to finding by chance owing to a number of scales being tested in the analysis in turn leading to spurious significant findings and also a lack of power to detect significant findings due to low sample size.

## Conclusions

In conclusion, this study identified clusters of variables that have previously been identified as determinants of HRQOL and survival in oesophageal cancer patients, which grouped patients into three distinct profiles. Howevere, none of these profiles could explain the substantial deterioration in HRQOL observed in the sub-sample of patients. Compared to the reference profile, patients in the adenocarcinoma profile had more constipation and dyspnoea, while patients in the squamous-cell carcinoma profile were more likely to deteriorate in constipation and trouble swallowing. These findings do not merit any clinical recommendations, but the search for the reasons for strong deterioration in HRQOL in patients who have been cured for oesophageal cancer must continue.
